# A new survival prediction model and exploration of hemodialysis quality control indicators in incident hemodialysis patients

**DOI:** 10.1371/journal.pone.0340994

**Published:** 2026-01-21

**Authors:** Huaiwen Chang, Xuehui Sun, Jing Qian, Li Ni, Ping Cheng, Jun Shi, Chuhan Lu, Xiaofeng Wang, Mengjing Wang, Jing Chen

**Affiliations:** 1 Division of Nephrology, Huashan Hospital, Fudan University, Shanghai, China; 2 Department of Computational Biology, School of Life Sciences, Fudan University, Shanghai, China; 3 Human Phenome Institute, Fudan University, Shanghai, China; 4 National Clinical Research Center for Aging and Medicine, Huashan Hospital, Fudan University, Shanghai, China; Japanese Red Cross Medical Center, JAPAN

## Abstract

**Objective:**

To develop and internally validate a Cox model predicting 1.5-year adverse outcomes (cardiovascular admission or all-cause mortality) in incident hemodialysis (HD) patients by integrating routinely recorded dialysis-machine parameters with traditional indicators.

**Methods:**

We retrospectively analyzed 74 incident end-stage renal disease (ESRD) patients who commenced thrice-weekly HD at Huashan Hospital, Fudan University, between 2012 and 2018. A total of 83 candidate variables, including demographics, traditional indicators (Kt/V, phosphorus, parathyroid hormone [PTH], albumin, hemoglobin, ultrafiltration volume), and dialysis machine parameters, were evaluated. Univariable and multivariable Cox regression identified predictors of 1.5-year outcomes.

**Results:**

The mean (± SD) age of the study population was 62 ± 14 years, and 55.4% were male. Independent predictors included serum alkaline phosphatase (ALP) measured at month 3 and machine-derived bicarbonate conductivity (BC) at month 6. A model combining ALP (month 3), bicarbonate conductivity (month 6), and traditional indicators (month 6) showed strong discrimination (AUC = 0.82). Achieving targets in ≥5 of 8 indicators—including ALP and BC—was associated with significantly better outcomes (log-rank *p* = 0.018).

**Conclusion:**

Integrating ALP and machine-derived BC into a Cox model significantly improves risk stratification in incident HD patients and facilitates the implementation of automated quality control.

## Introduction

The number of incident hemodialysis patients is increasing globally [1]. Hemodialysis is a life-saving therapy for patients with advanced uremia. However, despite its widespread adoption, the initial phase of hemodialysis is associated with significant dangers, particularly manifesting as excessive cardiovascular admissions and mortality, especially during the first year of treatment [2,3]. Recent studies on incident hemodialysis patients have reported a first-year mortality rate as high as 20% [4,5]. Given the high medical and care costs, early identification of high-risk incident hemodialysis patients is critical for clinical decision-making and poses a challenge to researchers and clinicians.

The management of dialysis patients has historically relied on a set of established quality control indicators, including Kt/V, serum albumin, hemoglobin, phosphorus, parathyroid hormone, and ultrafiltration volume. These indicators are likewise applied to incident hemodialysis patients. While these traditional markers offer valuable insights, the dynamic nature of incident patients during the early stages of dialysis may not be fully captured, and frequent testing could impose an increased clinical burden. Consequently, the predictive capabilities of models that utilize these traditional indicators have been observed to vary across different studies. On the one hand, these indicators may lack the capacity to adequately reflect the dynamic changes observed in new dialysis patients; on the other hand, the incorporation of additional markers could potentially enhance the prediction of dialysis outcomes. Exploratory efforts have been made to identify non-routine markers [6], but their practicality remains limited, with predictive power similar to that of routine clinical indicators.

During hemodialysis, a variety of parameters are continuously and automatically collected. These parameters include blood flow, ultrafiltration, dialysate flow rates, vessel pressure, temperature, and levels of bicarbonate and sodium. These machine parameters, informed by clinical knowledge, are potentially associated with various indicators known to influence patient outcomes. For instance, parameters such as dialysate flow rate, dialysis duration, and the type of dialyzer used may offer insights into urea clearance efficiency, a key factor in dialysis adequacy. A number of studies have indicated that arterial and venous pressures may serve as predictors of cardiovascular metrics, including blood pressure, heart rate, and cardiac electrical activity. These metrics have been demonstrated to exhibit a correlation with cardiovascular events [7–9]. Furthermore, an association has been demonstrated between sodium concentration in the dialysate and alterations in fluid volume among dialysis patients [10].

Consequently, the incorporation of these machine parameters may offer supplementary insights into the assessment of cardiovascular health, toxin clearance, and fluid balance. While traditional clinical indicators remain paramount in the oversight of dialysis quality, the incorporation of dialysis machine parameters holds the potential to augment the predictive capabilities. Additionally, the incorporation of continuously collected parameters has the potential to reduce the financial burden associated with frequent biochemical tests. However, further research is warranted to explore the full potential of machine-derived parameters in predicting long-term outcomes and improving dialysis care.

The objectives of this study were twofold: (i) to assess the potential association of demographic characteristics, traditional dialysis quality indicators, and hemodialysis machine parameters with the risk of all-cause death or cardiovascular disease admission in incident hemodialysis patients, and (ii) to generate a prediction model that may initially establish a more suitable dialysis quality control standard for incident hemodialysis patients.

## Materials and methods

### Study population

We retrospectively included 74 adults with end-stage renal disease (ESRD) who initiated thrice-weekly hemodialysis at Huashan Hospital between 03/02/2012 and 15/03/2018. Source data from the hospital’s electronic medical records, laboratory information system, and dialysis machine logs were accessed for research on 17/12/2022; the final analytic dataset was frozen (data lock) on 28/10/2023.

The inclusion criteria for the study were as follows: First, patients with end-stage renal disease requiring hemodialysis therapy were included, with no previous dialysis prior to enrollment. Secondly, the study population included patients aged 18 years or older. Thirdly, patients receiving hemodialysis for a period exceeding six months were included in the study. The following criteria were used to exclude subjects from the study: First, patients who had undergone living kidney transplantation within three months or conversion to peritoneal dialysis were excluded from the study. Secondly, patients who were pregnant or actively planning to become pregnant within the subsequent 24 months were excluded from the study.

Patients received standardized thrice-weekly hemodialysis. Single-pool Kt/V was calculated using Daugirdas’ second-generation formula [11]. Primary kidney disease was classified as chronic glomerulonephritis, diabetic nephropathy, hypertensive nephrosclerosis, polycystic kidney disease, or other/unknown, in line with the categories used in the Chinese Dialysis Outcomes and Practice Patterns Study (DOPPS5, 2012–2015) [12]. Comorbidities were systematically ascertained from the medical records, including diabetes mellitus, hypertension, cardiovascular disease, and a history of malignancy. This study was approved by the Ethics Committee of Huashan Hospital, Fudan University (KY2021−584). Written informed consent was obtained from all participants prior to enrollment. All participants were adults (≥18 years); therefore, parental or guardian consent was not applicable. All procedures were conducted in accordance with the Declaration of Helsinki and relevant institutional guidelines.

The term “high-risk” was defined as patients who experienced cardiovascular disease admission or all-cause mortality during the 1.5-year follow-up period. Individuals who did not require hospitalization or were hospitalized for reasons unrelated to cardiovascular disease (e.g., infection, vascular access revision, or other non-cardiovascular issues) were classified into the “low-risk” group. In instances where multiple diagnoses were documented (e.g., heart failure accompanied by pulmonary infection), the principal admission cause ascribed by the attending physician served as the determiner of group allocation.

### Biochemical assays and other measurements

Blood samples were obtained prior to the commencement of dialysis treatment. Serum biomarkers were subsequently measured using traditional laboratory methods, which included the assessment of sodium, potassium, calcium, phosphorus, chloride, magnesium, serum albumin, carbon dioxide binding capacity, alkaline phosphatase (ALP), hemoglobin, glycosylated hemoglobin, parathyroid hormone (PTH), ferritin, serum creatinine, and blood urea nitrogen (BUN). Subsequent measures were obtained at the third and sixth months of enrollment (denoted M3/M6) to capture dynamic changes in early dialysis. The parameters of the dialysis machine included dialysate parameters (conductivity, dialysate flow rate, etc.), ultrafiltration parameters (ultrafiltration volume, treatment time, etc.), pressure ranges (venous pressure, transmembrane pressure, etc.), and heparin parameters (heparin rate, arterial needle type, etc.) (see S1 Table in S1 File for complete markers). Total body water (TBW) at month 3 was not measured directly but estimated using the Watson formula based on sex, age, height, and post-dialysis body weight. Serum carbon dioxide binding power (CBP) was measured in mmol/L as part of the routine serum chemistry panel in the hospital central laboratory and reflects total CO₂ content, predominantly bicarbonate.

The primary study outcomes of interest included cardiovascular disease admissions and all-cause mortality. Patients were observed over a two-year period, with detailed follow-up at 1.5 years, given that excessive new hemodialysis mortality often occurs within the first two years [13].

### Statistical analysis

A total of 83 features were collected, with an average missing rate of 8%. Missing values were imputed using iterative imputation methods based on random forest regression. Continuous variables are expressed as means (standard deviations), while categorical variables are expressed as counts (percentages). The patients were stratified into two groups based on their prognosis within 1.5 years: a high risk group, defined as all-cause death or cardiovascular disease admission, and a low risk group, defined as admission for other reasons or no admission. Statistical comparisons were conducted using the Wilcoxon rank sum test and the χ2 test.

In the initial phase of the study, factors influencing poor prognosis were examined using univariate Cox proportional hazards analysis. We then specified a primary multivariable Cox model that focused on a parsimonious set of predictors that are not part of the traditional evaluation metrics, namely ALP at month 3 and BC at month 6, with adjustment for age and sex [14]. As a sensitivity analysis, we also fitted an exploratory saturated multivariable Cox model that included all predictors with *p* values less than 0.05 in univariable analyses together with age and sex; the hazard ratio estimates for this saturated model are presented in S4 Table in S1 File. Continuous predictors were z-standardized (per 1 SD increase) before Cox regression; hazard ratios therefore represent the effect per SD increment. To evaluate potential multicollinearity, we examined pairwise correlation coefficients and calculated variance inflation factors for candidate predictors.

The discriminatory power of these risk factors was assessed by generating receiver operating characteristic (ROC) curves, using bootstrap resampling with 1,000 iterations for internal validation, and recording accuracy, sensitivity, specificity, F_1_ score, and area under the curve (AUC, 95% CI). The optimal cutoff was selected by maximizing the Youden index at 1.5 years. Pairwise comparisons of AUCs between models were performed using DeLong’s test.

In order to make a comparison of the discriminatory ability of significant predictors for poor prognosis, each predictor’s value was converted into a score, the scores were summed, and the total score was classified as a binary variable. Subsequent to this, Kaplan-Meier plots and log-rank tests were utilized for further assessment. Finally, the predictive model was visualized through the implementation of column-line plots (nomograms). All analyses were conducted on the total sample. A two-tailed *p*-value less than 0.05 was considered statistically significant. All analyses were executed in R (version 4.1.2), with the exception of missing value imputation and certain hypothesis tests, which utilized Python 3.9 and Scikit-learn [15].

## Results

### Baseline characteristics

Table 1 summarizes baseline characteristics of the 74 incident hemodialysis patients and compares the low risk (n = 60) and high risk groups (n = 14). The complete characteristics are presented in S2 Table in S1 File. The demographic data indicate that 41 patients (55.4%) were male and 33 (44.6%) female, with a mean age of 62 ± 14 years. No significant differences in age or sex distribution were observed between groups. The low-risk group demonstrated a mean total body water (TBW) of 32.6 L, while the high-risk group exhibited a mean TBW of 36.7 L. Furthermore, the Kt/V ratio exhibited a higher value in the low-risk group (1.4) compared to the high-risk group (1.2). A higher proportion of patients in the low-risk group utilized an arteriovenous fistula (80%) compared to a catheter (20%), while the high-risk group exhibited 60% fistula vs. 40% catheter usage. The leading causes of end-stage renal disease were chronic glomerulonephritis (n = 27, 36.5%), diabetic nephropathy (n = 18, 24.3%), hypertensive nephrosclerosis (n = 10, 13.5%), polycystic kidney disease (n = 8, 10.8%), and other or unknown causes (n = 11, 14.9%). The most frequent comorbidities at baseline were hypertension (n = 25, 33.8%), cardiovascular disease (n = 22, 29.7%), diabetes mellitus (n = 14, 18.9%), and a history of malignancy (n = 9, 12.2%).

**Table 1:** Baseline characteristics of incident hemodialysis patients.

	All Patients	Lower risk group	Higher risk group	*P*
	(*n* = 74)	(*n* = 60)	(*n* = 14)	
Demographics				
Age, yr	62.1 (13.9)	62.9 (12.8)	58.4 (18.2)	0.746
Sex				0.101
Male	41 (0.6)	30 (0.5)	11 (0.8)	
Female	33 (0.4)	30 (0.5)	3 (0.2)	
Height, cm	164.7 (7.8)	164.2 (7.8)	166.6 (7.9)	0.337
Post-dialysis Weight (M3), kg	59 (11.6)	57.8 (9.5)	64.2 (17.7)	0.327
Total body water (M3), L	33.4 (5.6)	32.6 (4.6)	36.7 (8.1)	0.090
BMI (M3), kg/m^2^	22.6 (3.7)	22.3 (3.3)	23.9 (5.2)	0.276
RRF, ml/min/1.73m^2^	1.76 (2.41)	1.82 (2.57)	1.51 (1.63)	0.825
Vascular Access				0.075
Arteriovenous fistula	58 (0.8)	50 (0.8)	8 (0.6)	
Catheter	16 (0.2)	10 (0.2)	6 (0.4)	
Single-pool Kt/V (M3)	1.4 (0.3)	1.4 (0.3)	1.2 (0.3)	0.051
Pre-dialysis SBP (M3), mm Hg	138.4 (19.7)	137.9 (20.1)	140.6 (18.6)	0.669
Pre-dialysis DBP (M3), mm Hg	80.1 (14.9)	80.2 (15.5)	79.4 (12.5)	0.820
Post-dialysis SBP (M3), mm Hg	136.2 (19.1)	136.3 (17.8)	135.5 (24.5)	0.679
Post-dialysis DBP (M3), mm Hg	81 (12.7)	80.5 (12.7)	83.2 (12.6)	0.460
Blood chemistry				
Hemoglobin (M3), g/L	97.4 (17.9)	98.2 (15.7)	94.1 (25.7)	0.095
Calcium (M3), mmol/L	2.2 (0.3)	2.2 (0.2)	2.3 (0.4)	0.847
Phosphorus (M3), mmol/L	1.6 (0.4)	1.6 (0.4)	1.6 (0.5)	0.831
Albumin (M3), g/L	38.4 (3.9)	38.6 (3.6)	37.5 (5)	0.521
Parathyroid hormone (M3), pg/mL	283.2 (216.8)	282.4 (232.2)	286.6 (139.3)	0.614
CBP (M3), mmol/L	21.7 (3.3)	21.6 (3.4)	22.1 (2.9)	0.590
Pre-dialysis creatinine (M3), µmol/L	798.9 (209.1)	793.1 (202.2)	823.9 (243.4)	0.423
Post-dialysis creatinine (M3), µmol/L	302.6 (104.3)	284.4 (90.1)	380.7 (127.3)	0.004
Pre-dialysis Urea nitrogen (M3), mmol/L	26.1 (6.5)	25.9 (6.5)	27 (6.9)	0.644
Post-dialysis Urea nitrogen (M3), mmol/L	8.8 (3.1)	8.2 (2.4)	11.4 (4.2)	0.006
Hemodialysis Equipment Treatment Parameters				
Treatment time (M3), hours	3.9 (0.3)	4.0 (0.3)	3.8 (0.3)	0.087
Reinfusion volume (M3), mL	261.5 (66.5)	268.7 (60.9)	230.7 (82.1)	0.061
Ultrafiltration volume (M3), mL	2056.9 (1017)	1978.5 (1070.6)	2392.9 (675.3)	0.065
Venous pressure (M3), mm Hg	101 (31.1)	103.5 (31.7)	90.4 (26.6)	0.134
Arterial pressure (M3), mm Hg	−90.8 (48.7)	−89.6 (52.2)	−95.9 (30.3)	0.619
Actual MTP (M3), mm Hg	113.6 (80.9)	114.2 (88)	110.9 (40.6)	0.629
Blood flow (M3), mL/min	142.8 (14.5)	143.5 (15)	139.9 (11.9)	0.440

Continuous variables are expressed as means (standard deviations). Categorical variables are expressed as counts (proportions). Total body water was calculated using the Watson formula (1980). BMI, body mass index; RRF, residual renal function; Kt/V, based on Daugirdas second generation logarithmically estimated single pool variable volume formula; CBP, carbon dioxide binding power (routine serum total carbon dioxide, predominantly bicarbonate); SBP, systolic blood pressure; DBP, diastolic blood pressure; BC, bicarbonate conductivity; MTP, maximum transmembrane pressure; M3, the third month after initiation of hemodialysis; M6, the sixth month after initiation of hemodialysis; Lower risk group, other reasons for hospitalization or non-hospitalization; Higher risk group, hospitalization for cardiovascular disease or all-cause death. *P* indicates the significance level of the hypothesis test (Wilcoxon rank sum test for numeric variables and χ^2^ test for categorical variables, two-tailed *p*-value < 0.05 was considered statistically significant).

Several indicators showed marked between-group differences at M3. In particular, post-dialysis creatinine (380.7 vs. 284.4 µmol/L) and post-dialysis urea nitrogen (11.4 vs. 8.2 mmol/L) were significantly higher in the high risk group (*p* = 0.004 and *p* = 0.006, respectively). Treatment time (3.8 vs. 4.0 hours) and reinfusion volume (230.7 vs. 268.7 mL) were lower in the high risk group, whereas ultrafiltration volume was higher (2392.9 vs. 1978.5 mL), although these differences did not reach statistical significance. At M6, pre-dialysis parathyroid hormone (306.1 vs. 223.9 pg/mL), post-dialysis creatinine (385.6 vs. 311.6 µmol/L), and ultrafiltration volume (2662.2 vs. 2006.3 mL) were all higher in the high risk group, as summarized in S2 Table in S1 File.

During the 1.5-year follow-up, 14 of the 74 incident hemodialysis patients (18.9%) experienced the primary outcome of all-cause death or cardiovascular hospitalization. Among these 14 patients, 6 died and 8 had at least one cardiovascular admission. Cardiovascular events included 3 cases of coronary artery disease, 2 cases of acute ischemic stroke, and 3 cases of intracerebral hemorrhage.

### Univariate and multivariate analysis with Cox proportional hazards models

Table 2 presents the association between baseline indicators and adverse prognosis. In univariable Cox analyses, ten variables were significantly associated with the primary outcome (*p* < 0.05): pre- and post-dialysis weight, total body water, Kt/V, ALP, post-dialysis urea nitrogen at M3, and Kt/V, BC, and ultrafiltration volume at M6. These variables mainly reflected dialysis adequacy and volume-related domains.

**Table 2:** Cox model hazard ratios (HRs) for adverse prognosis in incident hemodialysis patients.

	Model 1		Model 2	
	HR (95% CI)	*P*-value	HR (95% CI)	*P*-value
Pre-dialysis Weight (M3), kg	1.69 (1.04 - 2.73)	0.033	—	—
Post-dialysis Weight (M3), kg	1.67 (1.04 - 2.7)	0.035	—	—
Total body water (M3), L	2.06 (1.22 - 3.49)	0.007	—	—
Kt/V (M3)	0.54 (0.3 - 0.97)	0.04	—	—
Kt/V (M6)	0.52 (0.28 - 0.98)	0.042	—	—
**Alkaline phosphatase (M3), U/L**	**1.37 (1.08 - 1.74)**	**0.011**	**1.63 (1.21 - 2.19)**	**0.001**
Post-dialysis creatinine (M3), µmol/L	1.82 (1.21 - 2.74)	0.004	—	—
Post-dialysis Urea nitrogen (M3), mmol/L	2.01 (1.34 - 3.02)	<0.001	—	—
**Bicarbonate conductivity (M6), mS/cm**	**1.57 (1.15 - 2.13)**	**0.004**	**1.55 (1.12 - 2.16)**	**0.009**
Ultrafiltration volume (M6), mL	1.95 (1.08 - 3.52)	0.027	—	—

Model 1: univariate models; only significant variables are presented. Model 2: prespecified multivariable Cox model including alkaline phosphatase at month 3 and bicarbonate conductivity at month 6, adjusted for age and sex. Variance inflation factors were calculated to assess multicollinearity; all variance inflation factors in Model 2 were less than 2.0. *P* values less than 0.05 were considered statistically significant. Kt/V, based on Daugirdas second-generation logarithmically estimated single-pool variable volume formula; M3, initial hemodialysis stage 3 (the third month); M6, initial hemodialysis stage 6 (the sixth month). All continuous predictors were z-standardized prior to Cox regression; hazard ratios represent the effect per 1-SD increase. Units are provided for clinical interpretability.

In the primary multivariable model, we focused on a parsimonious set of clinically prespecified predictors that are not part of the traditional evaluation metrics, namely alkaline phosphatase at month 3 and bicarbonate conductivity at month 6, with adjustment for age and sex (Model 2 in Table 2). In this model, alkaline phosphatase at month 3 and bicarbonate conductivity at month 6 remained independently associated with adverse prognosis, with hazard ratios (HRs) of 1.63 (95% confidence interval [CI]: 1.21–2.19, *P* = 0.001) and 1.55 (95% CI: 1.12–2.16, *P* = 0.009), respectively. All variance inflation factors in the primary model were less than 2.0, indicating no important multicollinearity among these predictors. Variance inflation factors and hazard ratio estimates for the exploratory saturated model are presented in S3 and S4 Tables in S1 File.

### Comparison of prognostic predictive performance of new indicators with traditional dialysis quality indicators

**Fig 1:**
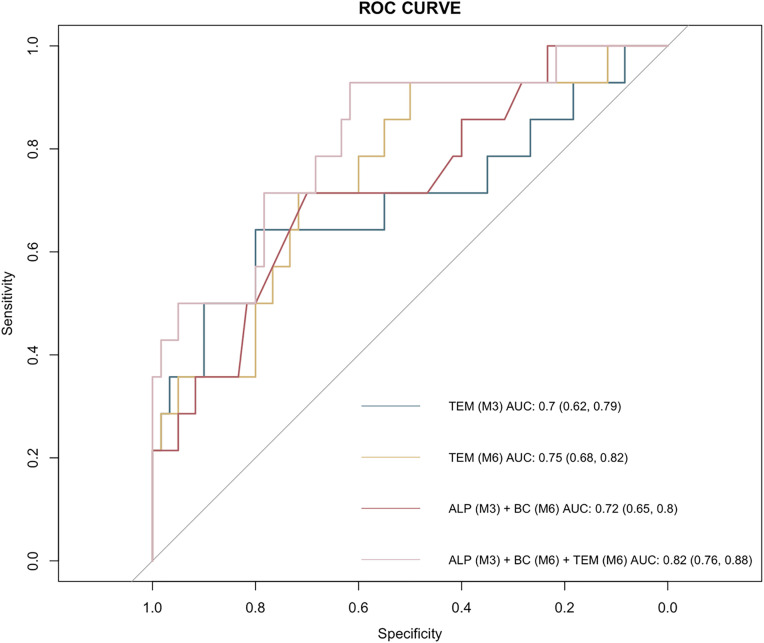
Receiver operating characteristic curve for estimating the discrimination of the multivariate Cox models. Receiver operating characteristic (ROC) curves show the predictive performance of traditional dialysis quality indicators (M3 or M6) and the combination of ALP (M3) and BC (M6), in isolation and in combination, for the risk of poor prognosis in patients on initial dialysis. The area under the curve (AUC) with 95% confidence intervals (CIs) was 0.70 (0.62–0.79) for traditional dialysis quality indicators (M3), 0.75 (0.68–0.82) for traditional dialysis quality indicators (M6), 0.72 (0.65–0.80) for the combination of ALP (M3) and BC (M6), and 0.82 (0.76–0.88) for the combined model including ALP (M3), BC (M6), and traditional indicators (M6). In pairwise comparison using DeLong’s test, the combined model showed significantly better discrimination than the model including traditional indicators (M6) alone (AUC 0.82 vs. 0.75; ΔAUC = 0.07; *p* = 0.012).

The present study evaluated the combined predictive performance of ALP (M3) and BC (M6), comparing it against traditional dialysis quality indicators (M3/M6) (Table 3), using ROC analysis with bootstrap 95% confidence intervals. The area under the curve (AUC) for traditional indicators (M3) was 0.70 (95% CI 0.62–0.79), while for M6 it was 0.75 (95% CI 0.68–0.82). Concurrently, ALP (M3) and BC (M6) exhibited an AUC of 0.72 (95% CI 0.65–0.80). Notably, incorporating ALP (M3), BC (M6), and the traditional indicators (M6) yielded the maximum AUC (0.82; 95% CI 0.76–0.88), signifying an enhanced discriminatory capacity (Fig 1). In pairwise comparisons using DeLong’s test, the combined model including ALP (M3), BC (M6), and traditional indicators (M6) demonstrated a statistically significant improvement in discrimination compared with the model including traditional indicators (M6) alone (AUC 0.82 vs. 0.75; ∆AUC = 0.07; *p* = 0.012).

**Table 3:** Comparison of the predictive performance of traditional dialysis quality indicators and combination of ALP (M3) and BC (M6).

	Accuracy	Sensitivity	Specificity	*F*_1_	AUC
Traditional dialysis quality indicators (M3)	0.67 (0.64, 0.71)	0.64 (0.62, 0.68)	0.80 (0.76, 0.82)	0.76 (0.73, 0.79)	0.70 (0.62, 0.79)
Traditional dialysis quality indicators (M6)	0.71 (0.65, 0.75)	0.71 (0.65, 0.74)	0.72 (0.69, 0.74)	0.80 (0.75, 0.83)	0.75 (0.68, 0.82)
ALP (M3) + BC (M6)	0.71 (0.69, 0.74)	0.71 (0.68, 0.77)	0.70 (0.68, 0.73)	0.80 (0.75, 0.83)	0.72 (0.65, 0.80)
ALP (M3) + BC (M6) + Traditional dialysis quality indicators (M6)	0.87 (0.82, 0.90)	0.93 (0.91, 0.94)	0.62 (0.60, 0.66)	0.92 (0.90, 0.96)	0.82 (0.76, 0.88)

Traditional dialysis quality indicators include Kt/V, blood phosphorus, parathyroid hormone, albumin, and hemoglobin; Kt/V, based on Daugirdas second generation logarithmically estimated single pool variable volume formula; ALP, alkaline phosphatase; BC, bicarbonate conductivity; AUC, area under the receiver operating characteristic (ROC) curve; CI, confidence interval; M3, initial hemodialysis stage 3 (the third month after initiation of dialysis); M6, initial hemodialysis stage 6 (the sixth month after initiation of dialysis). The confidence intervals provided were obtained using the Bootstrap method. Pairwise comparisons of AUCs were performed using DeLong’s test; details are provided in the Results section.

**Fig 2:**
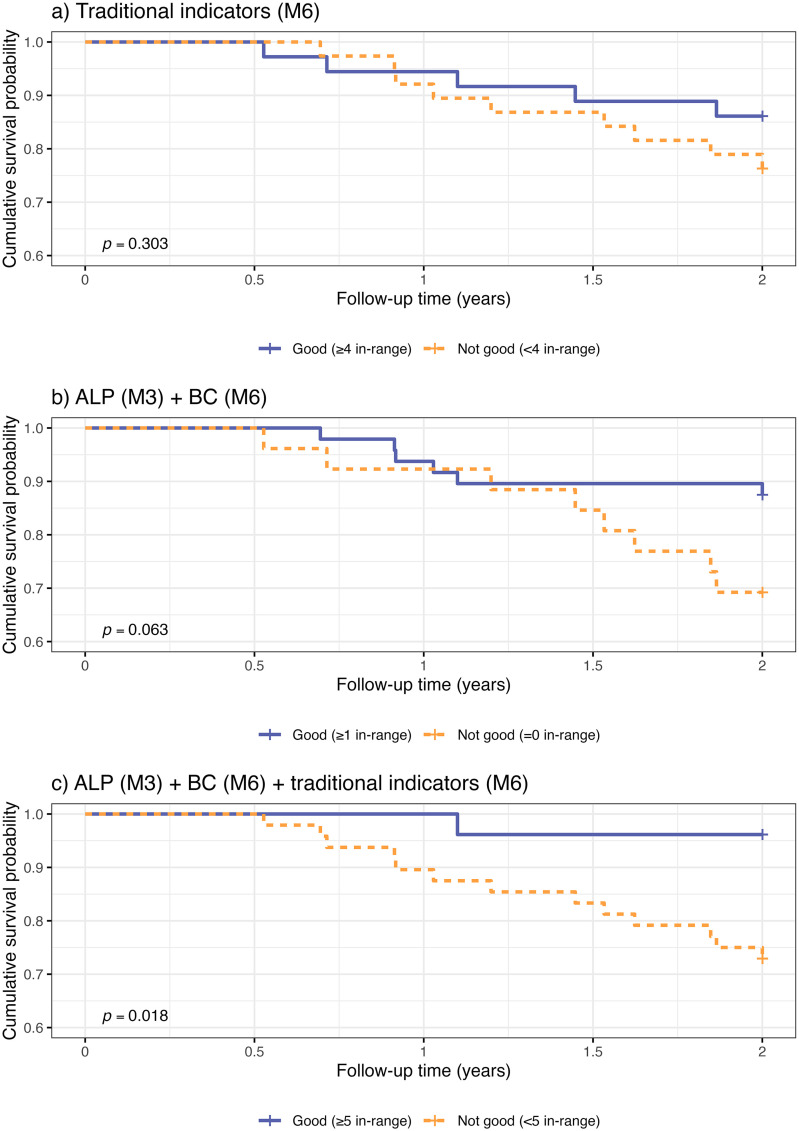
Kaplan-Meier analysis plots of binary variables for traditional dialysis quality indicators (TEM, M6), ALP (M3) and BC (M6). Cumulative survival curves of initial hemodialysis patients according to **a** traditional dialysis quality indicators (M6) group (Good [≥ 4] versus Not good [< 4]); **b** ALP (M3) + BC (M6) group (Good [≥ 1] versus Not good [= 0]); **c** ALP (M3) + BC (M6) + TEM (M6) group (Good [≥ 5] versus Not good [< 5]).

**Fig 3:**
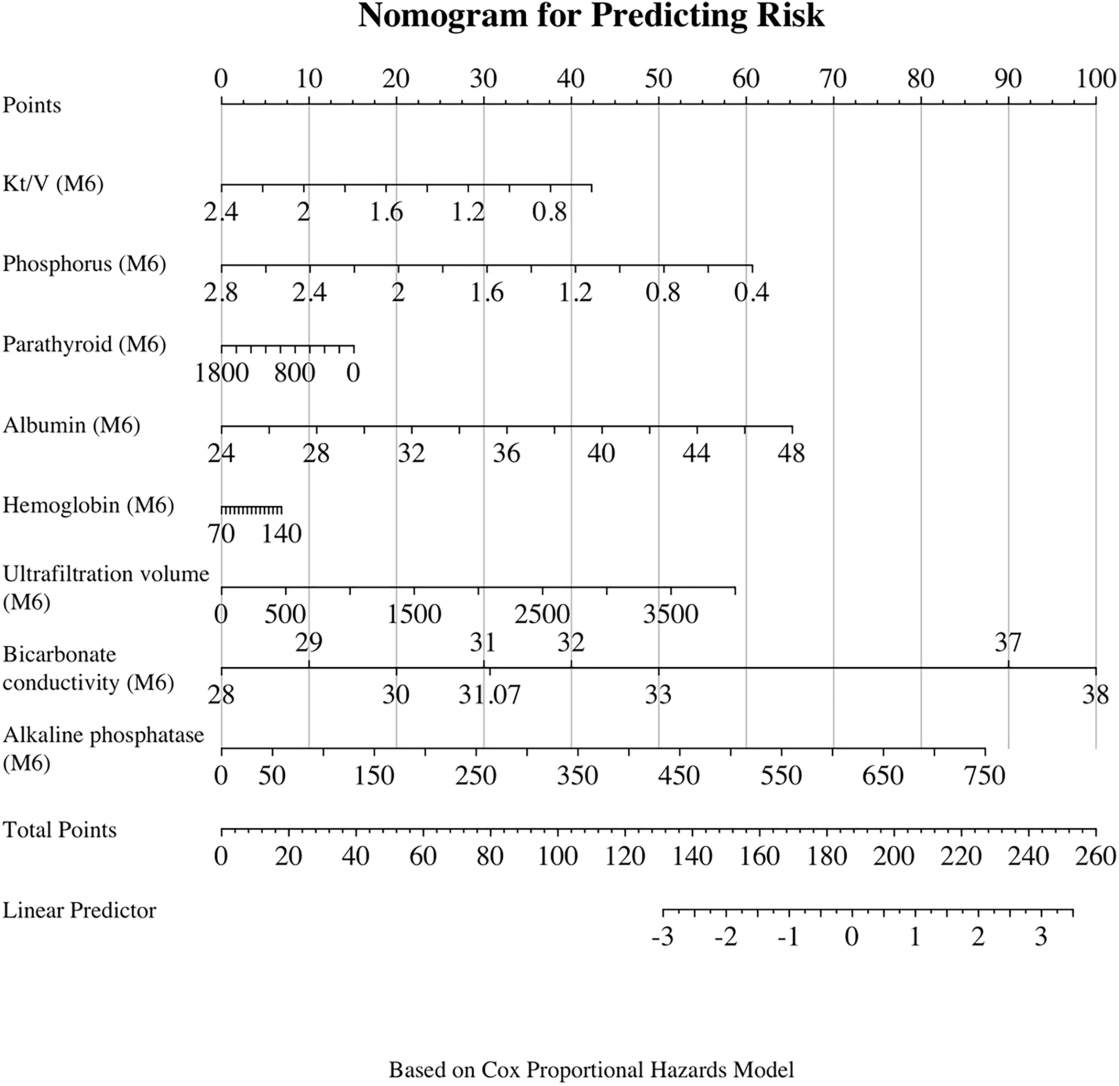
Cox model-based nomogram illustrating the relationship between traditional dialysis quality indicators (M6), ALP (M3), and BC (M6). For each patient, the value of each predictor is identified on its corresponding axis, and a vertical line is drawn upward to the “Points” axis to determine the score for that predictor. The cumulative score across all predictors is then converted into a total risk score, which corresponds to a linear predictor that estimates the patient’s risk of a poor prognosis.

To illustrate this, we defined clinical target ranges (Table 4) for each indicator [16–19], then categorized them into dichotomous variables and performed Kaplan-Meier analysis (Fig 2 and Table 5). The analysis indicated that the presence of four or more of the six traditional indicators within the acceptable range did not adequately predict adverse outcomes (log-rank *p* = 0.303). However, the incorporation of ALP (M3) and BC (M6) as supplementary indicators (resulting in a total of eight) demonstrated that patients with at least one of these two indicators within the acceptable range tended to have better outcomes, although this difference did not reach conventional statistical significance (log-rank *p* = 0.063). By contrast, when all eight indicators were considered together, patients with five or more indicators within the acceptable range exhibited significantly improved outcomes (log-rank *P* = 0.018). Furthermore, the nomogram-based scoring method (Fig 3) underscored the pivotal roles of ALP (M3) and BC (M6) in determining overall risk, thereby emphasizing their significance in the early stages of hemodialysis.

**Table 4:** Clinical significance and range of traditional dialysis quality indicators (M6), ALP (M3) and BC (M6) and patient assessment.

Marker	Significance	Range	Within target	Out of target
Traditional hemodialysis quality indicators (M6)				
Urea	Low molecular toxins	Kt/V ≥ 1.2	54 (0.73)	20 (0.27)
Phosphorus	Cardiovascular Diseases	1.13–1.78 mmol/L	40 (0.54)	34 (0.46)
PTH	Medium molecular toxins Metabolic bone disease	150–300 pg/mL	24 (0.32)	60 (0.68)
Albumin	Nutritional status	≥ 40 g/L	39 (0.53)	35 (0.47)
Hemoglobin	Anemia	110–130 g/L	27 (0.36)	47 (0.64)
UV	Body fluid balance	≤ 13 × W × T mL	45 (0.61)	29 (0.39)
New hemodialysis quality indicators				
ALP (M3)	CKD-Mineral and Bone Disorder	< 80 U/L	45 (0.61)	29 (0.39)
BC (M6)	Acid-base balance	< 3.1 mS/cm	9 (0.12)	65 (0.88)

Within target, the corresponding indicators fall within the standard clinical range, expressed as cases (%); out of target, the corresponding indicators are outside the standard clinical range, expressed as cases (%); PTH, Parathyroid hormone; UV, Ultrafiltration volume; W, Weight after dialysis (per patient); T, Time for dialysis (per patient); Kt/V, based on Daugirdas second generation logarithmically estimated single pool variable volume formula; ALP, alkaline phosphatase; BC, bicarbonate conductivity; M3, the third month after initiation of hemodialysis; M6, the sixth month after initiation of hemodialysis.

**Table 5:** Combined effect of traditional hemodialysis quality indicators (M6), ALP (M3) and BC (M6) on poor prognosis in incident hemodialysis patients: a Kaplan-Meier analysis.

	Meaning	Case (%)	*P*
Traditional hemodialysis quality indicators (M6), total of 6 indicators			0.303
Good	4 and more indicators in range	36 (0.49)	
Not good	Less than 4 indicators in range	38 (0.51)	
ALP (M3) + BC (M6), total of 2 indicators			0.063
Good	1 or 2 indicators in range	48 (0.65)	
Not good	No indicators in range	26 (0.35)	
ALP (M3) + BC (M6) + Traditional hemodialysis quality indicators (M6), total of 8 indicators			0.018
Good	5 and more indicators in range	26 (0.35)	
Not good	Less than 5 indicators in range	48 (0.65)	

ALP, alkaline phosphatase; BC, bicarbonate conductivity; M3, the third month after initiation of hemodialysis; M6, the sixth month after initiation of hemodialysis. Log-rank test *P* < 0.05 was considered statistically significant.

## Discussion

Using Cox models, we identified ALP at month 3 and machine-derived bicarbonate conductivity at month 6 as independent predictors of adverse outcomes in incident HD patients. The combination of these two parameters with established dialysis quality indicators (Kt/V, serum phosphorus, parathyroid hormone, albumin, and hemoglobin) yielded a significant enhancement in the model’s discriminatory capability, resulting in an increased AUC value to 0.82. When five or more of these eight indicators were within target ranges, prognosis could be predicted with greater accuracy. These findings suggest that while traditional quality control indicators perform well in assessing patient outcomes, the inclusion of additional markers such as ALP and bicarbonate conductivity may further enhance predictive accuracy. The validation and translation of these findings into clinical practice could facilitate a more precise risk stratification of incident hemodialysis patients and contribute to the expansion of the current set of dialysis quality control indicators.

This study possesses certain advantages over previous research on poor prognosis of dialysis, primarily due to its foundation on a retrospective analysis of high-risk incident hemodialysis patients. In the preliminary stage, strict judgments and requirements were made regarding the enrollment conditions of patients. Additionally, a more comprehensive set of parameters for dialysis machines was developed to predict poor prognoses in incident hemodialysis patients. Traditionally, Mandrekar et al. have proposed that, in the context of receiver operating characteristic curves in diagnostic test evaluation, AUC values greater than 0.70 are deemed acceptable, and values exceeding 0.80 are regarded as excellent. The Cox regression model that was constructed demonstrated convergent excellent predictive performance (AUC = 0.82; 95% CI 0.76, 0.88), which was comparatively higher than the predictive models that were previously constructed for predicting the risk of poor prognosis in incident hemodialysis patients, with AUC = 0.75 for the random forest model of Akbilgic et al. In previous studies, the AUCs for the four equations of Prouvot et al. and Yang et al.‘s CHADS2 score is 0.61-0.70, while the AUC for Quinn et al.’s logistic regression model is 0.76 [20–23]. This discrepancy can be attributed to the inherent limitations of traditional Cox regression models, which utilize complete survival time ending data without accounting for the inherent characteristics of the time distribution. Beyond the disparities in the applied prediction methods, the observed discrepancies can be attributed to the heterogeneity of the study patient populations and the variation in the available predictor variables.

The present findings align with the observations of other studies, which demonstrated that elevated ALP and BC levels are associated with a poor prognosis in hemodialysis patients [24–27]. Of particular significance is the observation of the satisfactory combined predictive utility of ALP (M3) and BC (M6) in incident hemodialysis patients. Previous studies have mainly focused on the elevated mortality rate among new hemodialysis patients [23,24]. However, the present study also encompassed the adverse prognostic implications of cardiovascular disease hospitalization. ALP, a membrane-bound enzyme, plays a pivotal physiological role in bone metabolism in patients with CKD [24]. Elevated ALP levels have been demonstrated to serve as a substantial predictor of augmented risk for high-turnover bone disease, a condition associated with ectopic calcification and an elevated risk of cardiovascular disease [28]. Furthermore, ALP consistently and independently predicts mortality [27]. Conversely, it is imperative to acknowledge that effective control of the bicarbonate concentration of dialysis fluid by BC is paramount to ensure acid-base balance in hemodialysis patients [17]. A substantial body of research has established a correlation between elevated bicarbonate levels in serum and suboptimal nutritional status, as well as diminished survival outcomes [29,30]. It is imperative for clinicians to exercise caution when administering bicarbonate to correct metabolic acidosis, particularly in cases of severe acidosis. Elevated bicarbonate concentrations, as seen in bicarbonate therapy, have been demonstrated to lack therapeutic benefit and may even increase mortality [31–34]. There is a broad consensus in the scientific community that the metabolism of bone tissue and the balance of acidity within the body significantly impact the prognosis of hemodialysis patients. To a certain extent, the maintenance or improvement of bone metabolism and acid-base balance has been demonstrated to reduce cardiovascular admissions and enhance survival outcomes [35,36].

When four or more of the six traditional dialysis quality indicators were within target ranges, they did not adequately predict poor prognosis in patients. However, the integration of ALP and bicarbonate conductivity (BC) into the traditional dialysis quality indicators led to a significant enhancement in prognostic predictions, with the attainment of 5 or more of the 8 indicators within target ranges. The established dialysis quality indicators, encompassing Kt/V, blood phosphorus, parathyroid hormone, albumin, and hemoglobin, are widely recognized and employed in Chinese hospitals as a standardized clinical reference. These indicators play a pivotal role in the management of hemodialysis centers and ensuring patient safety [37,38]. However, the findings of this study suggest that the incorporation of ALP (M3) and BC (M6) may offer enhanced prognostic risk differentiation, particularly in the context of prolonged patient monitoring (1.5 years). This expanded set of markers could assist clinicians in refining their understanding of dialysis adequacy and identifying a greater number of patients at high risk, facilitating timely interventions. Moreover, the integration of these supplementary markers with traditional quality indicators enhanced the model’s discriminatory capability (AUC = 0.82), thereby offering a more comprehensive approach to risk stratification. These cost-effective and readily implementable screening methods hold considerable potential to enhance the identification of high-risk incident hemodialysis patients and optimize patient care.

The present study has three main strengths. Firstly, to our knowledge, it is among the first studies to incorporate dialysis machine–derived parameters in incident hemodialysis patients to evaluate the risk of poor prognosis, thereby broadening the set of candidate indicators for dialysis quality control and proposing a practical quality-control tool (Table 5). Secondly, the combined model including ALP (M3), BC (M6), and traditional dialysis quality indicators (M6) demonstrated excellent discriminative performance (AUC = 0.82), supporting its potential use for early risk stratification and guidance of prevention and management in incident hemodialysis patients. Thirdly, the predictors identified in this study are simple, routinely measured clinical variables that are assessed at defined early time points (M3 and M6), providing temporal resolution of early dialysis adaptation rather than relying on long-term averages as in previous studies (Okuno et al. [39], Owen et al. [40]).

This study has several limitations. First, it was conducted in a single center with a relatively small sample and only 14 primary outcome events, which may limit generalizability [41]. Although we used bootstrap internal validation to quantify model optimism and obtain confidence intervals for the AUC, the absence of an external validation cohort means that the proposed model should be regarded as exploratory and hypothesis generating rather than ready for routine clinical use. Second, assessments at months 3 and 6 may not capture the full clinical trajectory. However, these time points are commonly used to monitor early adaptation in new dialysis patients and are practical anchors for early risk stratification in routine care. Third, several baseline variables that were associated with outcomes in univariable analyses were not retained in the parsimonious multivariable model because of concerns about overfitting and collinearity. These candidate predictors, together with ALP and bicarbonate conductivity, will need to be reevaluated in larger multicenter cohorts with more events and longer follow-up.

## Conclusion

In summary, the present study identified the combination of ALP and BC as a significant predictor of poor prognosis in incident hemodialysis patients. Moreover, the integration of these measures with traditional dialysis quality indicators has the potential to enhance discrimination, thereby potentially improving dialysis quality. Further prospective studies with external validation cohorts are needed to substantiate and expand the clinical application of these findings. The objective of such studies would be to substantiate an augmented standard of dialysis quality control.

## Supporting information

S1 Checklist STROBE Statement checklist of items that should be included in reports of observational cohort studies. (DOCX)

S1 File Supplementary tables S1–S4 providing the list of markers used in this study (Table S1), detailed baseline characteristics of incident hemodialysis patients (Table S2), variance inflation factors for the exploratory saturated multivariable Cox model (Table S3), and hazard ratio estimates for the exploratory saturated multivariable Cox model including all candidate predictors (Table S4). (DOCX)

## References

[1] Himmelfarb J, Vanholder R, Mehrotra R, Tonelli M. The current and future landscape of dialysis. Nat Rev Nephrol. 2020;16(10):573–85. doi: 10.1038/s41581-020-0315-4 32733095 PMC7391926

[2] McIntyre CW, Rosansky SJ. Starting dialysis is dangerous: how do we balance the risk?. Kidney Int. 2012;82(4):382–7. doi: 10.1038/ki.2012.133 22534960

[3] Zhu Y, Lai Y, Hu Y, Fu Y, Zhang Z, Lin N, et al. The mechanisms underlying acute myocardial infarction in chronic kidney disease patients undergoing hemodialysis. Biomedicine & Pharmacotherapy. 2024;177:117050. doi: 10.1016/j.biopha.2024.117050 38968794

[4] Kang D-H, Streja E, You AS, Lee Y, Narasaki Y, Torres S, et al. Hypoglycemia and Mortality Risk in Incident Hemodialysis Patients. J Ren Nutr. 2024;34(3):200–8. doi: 10.1053/j.jrn.2023.09.001 37918644

[5] Bossola M, Di Napoli A, Angelici L, Bargagli AM, Cascini S, Kirchmayer U, et al. Trend and determinants of mortality in incident hemodialysis patients of the Lazio region. BMC Nephrol. 2023;24(1). doi: 10.1186/s12882-023-03170-w PMC10134676 37101132

[6] Shiotsu Y, Mori Y, Nishimura M, Hatta T, Imada N, Maki N, et al. Prognostic utility of plasma S100A12 levels to establish a novel scoring system for predicting mortality in maintenance hemodialysis patients: a two-year prospective observational study in Japan. BMC Nephrol. 2013;14(1). doi: 10.1186/1471-2369-14-16 PMC3552940 23324110

[7] Stewart J, Stewart P, Walker T, Horner DV, Lucas B, White K, et al. A Feasibility Study of Non-Invasive Continuous Estimation of Brachial Pressure Derived From Arterial and Venous Lines During Dialysis. IEEE J Transl Eng Health Med. 2020;9:2700209. doi: 10.1109/JTEHM.2020.3035988 33200053 PMC7665243

[8] Holmer M, Sandberg F, Solem K, Grigonyte E, Olde B, Sörnmo L. Extracting a cardiac signal from the extracorporeal pressure sensors of a hemodialysis machine. IEEE Trans Biomed Eng. 2015;62(5):1305–15. doi: 10.1109/TBME.2014.2385964 25546855

[9] Holmer M, Sandberg F, Solem K, Olde B, Sörnmo L. Cardiac signal estimation based on the arterial and venous pressure signals of a hemodialysis machine. Physiol Meas. 2016;37(9):1499–515. doi: 10.1088/0967-3334/37/9/1499 27511299

[10] Lee SW. Sodium balance in maintenance hemodialysis. Electrolyte Blood Press. 2012;10(1):1–6. doi: 10.5049/EBP.2012.10.1.1 23508564 PMC3597912

[11] Daugirdas JT. Second generation logarithmic estimates of single-pool variable volume Kt/V: an analysis of error. J Am Soc Nephrol. 1993;4(5):1205–13. doi: 10.1681/ASN.V451205 8305648

[12] Zhao X, Niu Q, Gan L, Hou FF, Liang X, Ni Z, et al. Thrombocytopenia predicts mortality in Chinese hemodialysis patients- an analysis of the China DOPPS. BMC Nephrol. 2022;23(1):11. doi: 10.1186/s12882-021-02579-5 34979949 PMC8722075

[13] Rosansky SJ, Eggers P, Jackson K, Glassock R, Clark WF. Early start of hemodialysis may be harmful. Arch Intern Med. 2011;171(5):396–403. doi: 10.1001/archinternmed.2010.415 21059968

[14] van Houwelingen AH, den Elzen WPJ, Mooijaart SP, Heijmans M, Blom JW, de Craen AJM, et al. Predictive value of a profile of routine blood measurements on mortality in older persons in the general population: the Leiden 85-plus Study. PLoS One. 2013;8(3):e58050. doi: 10.1371/journal.pone.0058050 23483967 PMC3587570

[15] Zhang F, Lu S, Tian M, Hu K, Chen R, Zhang B, et al. Albumin-to-Alkaline Phosphatase Ratio is an Independent Prognostic Indicator in Combined Hepatocellular and Cholangiocarcinoma. J Cancer. 2020;11(17):5177–86. doi: 10.7150/jca.45633 32742464 PMC7378922

[16] Bertocchio J, Mohajer M, Gaha K, Ramont L, Maheut H, Rieu P. Modifications to bicarbonate conductivity: A way to increase phosphate removal during hemodialysis? Proof of concept. Hemodialysis International. 2016;20(4):601–9. doi: 10.1111/hdi.12423 27060343

[17] Krahn RE, Tulowitzki R, Gudleski GD, Murray B, Rajagopalan B, Su W, et al. Effect of Bicarbonate-Buffered Dialysate on Ventricular Arrhythmias in Hemodialysis Patients. Am J Nephrol. 2019;49(1):74–80. doi: 10.1159/000495846 30602157

[18] Sargent JA, Yamamoto T, Yamakawa T, De Waal D, Gennari FJ. Hemodialysis using a low bicarbonate dialysis bath: Implications for acid-base homeostasis. Semin Dial. 2020;33(5):402–9. doi: 10.1111/sdi.12902 32798324

[19] Do C, Vasquez PC, Soleimani M. Metabolic Alkalosis Pathogenesis, Diagnosis, and Treatment: Core Curriculum 2022. Am J Kidney Dis. 2022;80(4):536–51. doi: 10.1053/j.ajkd.2021.12.016 35525634 PMC10947768

[20] Akbilgic O, Obi Y, Potukuchi PK, Karabayir I, Nguyen DV, Soohoo M, et al. Machine Learning to Identify Dialysis Patients at High Death Risk. Kidney Int Rep. 2019;4(9):1219–29. doi: 10.1016/j.ekir.2019.06.009 31517141 PMC6732773

[21] Prouvot J, Pambrun E, Antoine V, Couchoud C, Vigneau C, Roche S, et al. Low performance of prognostic tools for predicting death before dialysis in older patients with advanced CKD. J Nephrol. 2022;35(3):993–1004. doi: 10.1007/s40620-021-01180-1 34787796

[22] van Diepen M, Schroijen MA, Dekkers OM, Rotmans JI, Krediet RT, Boeschoten EW, et al. Predicting mortality in patients with diabetes starting dialysis. PLoS One. 2014;9(3):e89744. doi: 10.1371/journal.pone.0089744 24594735 PMC3942369

[23] Quinn RR, Laupacis A, Hux JE, Oliver MJ, Austin PC. Predicting the risk of 1-year mortality in incident dialysis patients: accounting for case-mix severity in studies using administrative data. Med Care. 2011;49(3):257–66. doi: 10.1097/MLR.0b013e318202aa0b 21301370

[24] Regidor DL, Kovesdy CP, Mehrotra R, Rambod M, Jing J, McAllister CJ, et al. Serum Alkaline Phosphatase Predicts Mortality among Maintenance Hemodialysis Patients. Journal of the American Society of Nephrology. 2008;19(11):2193–203. doi: 10.1681/asn.2008010014 18667733 PMC2573010

[25] Kitamura H, Yamada S, Hiyamuta H, Yotsueda R, Taniguchi M, Tokumoto M, et al. Serum Alkaline Phosphatase Levels and Increased Risk of Brain Hemorrhage in Hemodialysis Patients: The Q-Cohort Study. J Atheroscler Thromb. 2022;29(6):923–36. doi: 10.5551/jat.62885 34108341 PMC9174090

[26] Guo J, Zeng M, Zhang Y, Huang H, Yang G, Xu F, et al. Serum Alkaline Phosphatase Level Predicts Cardiac Valve Calcification in Maintenance Hemodialysis Patients. Blood Purif. 2020;49(5):550–9. doi: 10.1159/000505846 32050204

[27] Tentori F, Karaboyas A, Robinson BM, Morgenstern H, Zhang J, Sen A, et al. Association of dialysate bicarbonate concentration with mortality in the Dialysis Outcomes and Practice Patterns Study (DOPPS). Am J Kidney Dis. 2013;62(4):738–46. doi: 10.1053/j.ajkd.2013.03.035 23707043 PMC3832240

[28] Park JC, Kovesdy CP, Duong U, Streja E, Rambod M, Nissenson AR, et al. Association of serum alkaline phosphatase and bone mineral density in maintenance hemodialysis patients. Hemodial Int. 2010;14(2):182–92. doi: 10.1111/j.1542-4758.2009.00430.x 20345388 PMC5509753

[29] Dobre M, Yang W, Chen J, Drawz P, Hamm LL, Horwitz E, et al. Association of Serum Bicarbonate With Risk of Renal and Cardiovascular Outcomes in CKD: A Report From the Chronic Renal Insufficiency Cohort (CRIC) Study. American Journal of Kidney Diseases. 2013;62(4):670–8. doi: 10.1053/j.ajkd.2013.01.017 23489677 PMC3701754

[30] Navaneethan SD, Schold JD, Arrigain S, Jolly SE, Wehbe E, Raina R, et al. Serum bicarbonate and mortality in stage 3 and stage 4 chronic kidney disease. Clin J Am Soc Nephrol. 2011;6(10):2395–402. doi: 10.2215/CJN.03730411 21885787 PMC3359558

[31] Chauveau P, Rigothier C, Combe C. Con: Higher serum bicarbonate in dialysis patients is protective. Nephrol Dial Transplant. 2016;31(8):1226–9. doi: 10.1093/ndt/gfw255 27411724

[32] Marecek R, Kissling S, Teso AD, Vuignier Y, Wojtusciszyn A. Chronic metabolic acidosis in a patient with diabetes on maintenance haemodialysis: mind the anion gap. The Lancet. 2024;404(10452):570–1. doi: 10.1016/s0140-6736(24)01452-1 39127481

[33] Kunz JV, Hansmann H, Fähndrich M, Pigorsch M, Bethke N, Peters H, et al. Standard vs. carbone dioxide adapted kidney replacement therapy in hypercapnic ARDS patients: a randomized controlled pilot trial (BigBIC). Crit Care. 2024;28(1):198. doi: 10.1186/s13054-024-04979-z 38863072 PMC11167756

[34] Rachoin J-S, Weisberg LS, McFadden CB. Treatment of lactic acidosis: appropriate confusion. J Hosp Med. 2010;5(4):E1-7. doi: 10.1002/jhm.600 20394011

[35] Naber T, Purohit S. Chronic Kidney Disease: Role of Diet for a Reduction in the Severity of the Disease. Nutrients. 2021;13(9):3277. doi: 10.3390/nu13093277 34579153 PMC8467342

[36] Wieliczko M, Małyszko J. Acid-base balance in hemodialysis patients in everyday practice. Ren Fail. 2022;44(1):1090–7. doi: 10.1080/0886022X.2022.2094805 35793495 PMC9272922

[37] Torreggiani M, Fois A, Njandjo L, Longhitano E, Chatrenet A, Esposito C, et al. Toward an individualized determination of dialysis adequacy: a narrative review with special emphasis on incremental hemodialysis. Expert Rev Mol Diagn. 2021;21(11):1119–37. doi: 10.1080/14737159.2021.1987216 34595991

[38] Xu W, Yi L, Deng C, Zhao Z, Ran L, Ren Z, et al. Maternal periconceptional folic acid supplementation reduced risks of non-syndromic oral clefts in offspring. Sci Rep. 2021;11(1):12316. doi: 10.1038/s41598-021-91825-9 34112890 PMC8192944

[39] Okuno S, Ishimura E, Kohno K, Fujino-Katoh Y, Maeno Y, Yamakawa T, et al. Serum beta2-microglobulin level is a significant predictor of mortality in maintenance haemodialysis patients. Nephrol Dial Transplant. 2009;24(2):571–7. doi: 10.1093/ndt/gfn521 18799606

[40] Owen WF Jr, Lew NL, Liu Y, Lowrie EG, Lazarus JM. The urea reduction ratio and serum albumin concentration as predictors of mortality in patients undergoing hemodialysis. N Engl J Med. 1993;329(14):1001–6. doi: 10.1056/NEJM199309303291404 8366899

[41] Austin GI, Pe’er I, Korem T. Distributional bias compromises leave-one-out cross-validation. ArXiv. 2025. 10.1126/sciadv.adx6976 PMC12662204 41313770

