# Supplementary material for: A new survival prediction model and exploration of hemodialysis quality control indicators in incident hemodialysis patients
Source: PLoS One. 2026 Jan 21;21(1):e0340994. doi: 10.1371/journal.pone.0340994 (PMC12822944; doi:10.1371/journal.pone.0340994)
Supplement: S1 File — (DOCX) [file pone.0340994.s002.docx]

**Table S1. List of markers used in this study**

| **Demographic indicators** |
| --- |
| Age, sex, weight before dialysis (M3 & M6), weight after dialysis (M3 & M6), total body water (Watson 1980 formula, M3 & M6), BMI (M3 & M6), BSA (Dubois formula, M3 & M6), vascular access, Kt/V (M3 & M6), Kru |
| **Biomarkers** (M3 / M6) |
| Sodium, Potassium, Albumin, Calcium, Inorganic phosphorus, Chlorine, Magnesium, Carbon dioxide binding, Alkaline phosphatase, Hemoglobin, Glycosylated hemoglobin, Parathyroid hormone, Ferritin, pre-dialysis blood creatinine, post-dialysis blood creatinine, pre-dialysis urea nitrogen, post-dialysis urea nitrogen |
| **Hemodialysis Machine Clinical Parameters** (M3 / M6) |
| Pre-dialysis systolic blood pressure, pre-dialysis diastolic blood pressure, post-dialysis systolic pressure, post-dialysis diastolic blood pressure, treatment time, blood flow, end conductivity, bicarbonate conductivity, dialysate flow rate, dialysate temperature, reinfusion volume, dialyzer name, dialysate calcium concentration, ultrafiltration volume, venous pressure, arterial pressure, actual maximum transmembrane pressure |

BMI, body mass index; BSA, body surface area; Kt/V, based on Daugirdas second generation logarithmically estimated single pool variable volume formula; Kru, kidney urea clearance; M3, the third month after initiation of hemodialysis; M6, the six month after initiation of hemodialysis.

**Table S2. Characteristics of incident hemodialysis patients**

|  | **All Patients** | **Lower risk group** | | **Higher risk group** | | ***P*** | |  |
| --- | --- | --- | --- | --- | --- | --- | --- | --- |
|  | **(*n* = 74)** | **(*n* = 60)** | | **(*n* = 14)** | |  | |  |
| Demographics |  |  |  |  |  |  |  |  |
| Age, yr | 62.1 (13.9) | 62.9 (12.8) | | 58.4 (18.2) | | 0.746 | |  |
| Sex |  |  | |  | | 0.101 | |  |
| Male | 41 (0.6) | 30 (0.5) | | 11 (0.8) | |  | |  |
| Female | 33 (0.4) | 30 (0.5) | | 3 (0.2) | |  | |  |
| Height, cm | 164.7 (7.8) | 164.2 (7.8) | | 166.6 (7.9) | | 0.337 | |  |
| weight BD (M3), kg | 61.5 (12.2) | 60.1 (9.9) | | 67.2 (18.6) | | 0.279 | |  |
| Weight BD (M6), kg | 61.8 (12.6) | 60.8 (11) | | 65.8 (18.2) | | 0.508 | |  |
| Weight AD (M3), kg | 59 (11.6) | 57.8 (9.5) | | 64.2 (17.7) | | 0.327 | |  |
| Weight AD (M6), kg | 61 (12.7) | 59.9 (10.7) | | 65.8 (18.9) | | 0.553 | |  |
| Total body water (M3), l  1980 formula, l | 33.4 (5.6) | 32.6 (4.6) | | 36.7 (8.1) | | 0.090 | |  |
| Total body water (M6), l | 33.3 (5.5) | 32.7 (4.9) | | 35.7 (7.3) | | 0.185 | |  |
| BMI (M3), kg/m^2^ | 22.6 (3.7) | 22.3 (3.3) | | 23.9 (5.2) | | 0.276 | |  |
| BMI (M6), kg/m^2^ | 22.8 (4) | 22.6 (3.6) | | 23.8 (5.3) | | 0.605 | |  |
| BSA (M3), m^2^ | 0.1 (0) | 0.1 (0) | | 0.1 (0) | | 0.448 | |  |
| BSA (M6), m^2^ | 0.1 (0) | 0.1 (0) | | 0.1 (0) | | 0.408 | |  |
| RRF, ml/min/1.73m^2^ | 1.76 (2.41) | 1.82 (2.57) | | 1.51 (1.63) | | 0.825 | |  |
| SBP BD(M3), mm Hg | 138.4 (19.7) | 137.9 (20.1) | | 140.6 (18.6) | | 0.669 | |  |
| SBP BD (M6), mm Hg | 135 (19.3) | 134.8 (18.9) | | 135.6 (21.4) | | 0.619 | |  |
| DBP BD(M3), mm Hg | 80.1 (14.9) | 80.2 (15.5) | | 79.4 (12.5) | | 0.820 | |  |
| DBP BD(M6), mm Hg | 78.4 (13.3) | 77.7 (12.8) | | 81.5 (15.4) | | 0.279 | |  |
| SBP AD(M3), mm Hg | 136.2 (19.1) | 136.3 (17.8) | | 135.5 (24.5) | | 0.679 | |  |
| SBP AD(M6), mm Hg | 134.4 (19.6) | 135.2 (19.9) | | 131.3 (18.3) | | 0.341 | |  |
| DBP AD(M3), mm Hg | 81 (12.7) | 80.5 (12.7) | | 83.2 (12.6) | | 0.460 | |  |
| DBP AD(M6), mm Hg | 81.5 (13.2) | 81.6 (12.8) | | 81.1 (15.2) | | 0.978 | |  |
| Vascular Access |  |  | |  | | 0.075 | |  |
| Arteriovenous fistula | 58 (0.8) | 50 (0.8) | | 8 (0.6) | |  | |  |
| Catheter | 16 (0.2) | 10 (0.2) | | 6 (0.4) | |  | |  |
| Kt/V (M3) | 1.4 (0.3) | 1.4 (0.3) | | 1.2 (0.3) | | 0.051 | |  |
| Kt/V (M6) | 1.4 (0.3) | 1.4 (0.2) | | 1.2 (0.3) | | 0.040* | |  |
| Blood chemistry |  |  | |  | |  | |  |
| Sodium (M3), mmol/L  mmol/L | 139.5 | 139.7 (3.2) | | 138.7 (2.8) | | 0.344 | |  |
| Sodium (M6), mmol/L | 138.9 | 138.9 (3.1) | | 139.2 (4.2) | | 0.912 | |  |
| Potassium (M3), mmol/L | 4.7 | 4.7 (0.8) | | 4.8 (0.9) | | 0.735 | |  |
| Potassium (M6), mmol/L | 4.6 | 4.6 (0.9) | | 4.8 (0.9) | | 0.314 | |  |
| Albumin (M3), g/L | 38.4 | 38.6 (3.6) | | 37.5 (5) | | 0.521 | |  |
| Albumin (M6), g/L | 39.4 | 39.7 (3.5) | | 37.9 (6.5) | | 0.639 | |  |
| Calcium (M3), mmol/L | 2.2 | 2.2 (0.2) | | 2.3 (0.4) | | 0.847 | |  |
| Calcium (M6), mmol/L | 2.3 | 2.2 (0.2) | | 2.3 (0.5) | | 0.863 | |  |
| Inorganic phosphorus (M3), mmol/L | 1.6 | 1.6 (0.4) | | 1.6 (0.5) | | 0.831 | |  |
| Inorganic phosphorus (M6), mmol/L | 1.5 | 1.5 (0.5) | | 1.6 (0.7) | | 0.408 | |  |
| Chlorine (M3), mmol/L | 100.4 | 100.7 (3.9) | | 98.7 (4.6) | | 0.181 | |  |
| Chlorine (M6), mmol/L | 98.8 | 98.6 (4.5) | | 99.6 (4.7) | | 0.464 | |  |
| Magnesium (M3), mmol/L | 1.0 | 1 (0.1) | | 1 (0.1) | | 0.918 | |  |
| Magnesium (M6), mmol/L | 1.1 (0.1) | 1.1 (0.1) | | 1.1 (0.1) | | 0.852 | |  |
| CBP (M3), mmol/L | 21.7 (3.3) | 21.6 (3.4) | | 22.1 (2.9) | | 0.590 | |  |
| CBP (M6), mmol/L | 21.5 (3.5) | 21.7 (3.2) | | 20.6 (4.4) | | 0.159 | |  |
| Alkaline phosphatase (M3), U/L | 81.9 (77) | 71.6 (20.2) | | 126.3 (169.8) | | 0.117 | |  |
| Alkaline phosphatase (M6), U/L | 80.4 (89) | 72 (25.9) | | 116.6 (199.1) | | 0.788 | |  |
| Hemoglobin (M3), g/L | 97.4 (17.9) | 139.7 (3.2) | | 138.7 (2.8) | | 0.344 | |  |
| Hemoglobin (M6), g/L | 105.6 (13.7) | 104.8 (13.5) | | 109 (14.3) | | 0.362 | |  |
| Glycosylated hemoglobin (M3), g/L | 5.7 (0.8) | 5.8 (0.8) | | 5.6 (0.8) | | 0.664 | |  |
| Glycosylated hemoglobin (M6), g/L | 5.9 (0.9) | 5.9 (0.9) | | 6.1 (1.2) | | 0.730 | |  |
| Parathyroid hormone (M3), μg/L | 283.2 (216.8) | 282.4 (232.2) | | 286.6 (139.3) | | 0.614 | |  |
| Parathyroid hormone (M6), μg/L | 239.5 (242.2) | 223.9 (250.2) | | 306.1 (198.4) | | 0.081 | |  |
| Ferritin (M3), μg/L | 190.5 (153.9) | 191.8 (165.8) | | 184.9 (89.8) | | 0.456 | |  |
| Ferritin (M6), μg/L | 205.7 (183.2) | 208.6 (193.1) | | 193.6 (138.3) | | 0.804 | |  |
| Blood creatinine BD (M3), µmol/L | 798.9 (209.1) | 793.1 (202.2) | | 823.9 (243.4) | | 0.423 | |  |
| Blood creatinine BD (M6), µmol/L | 847.8 (262.2) | 838 (260.5) | | 889.6 (275.3) | | 0.314 | |  |
| Blood creatinine AD (M3), µmol/L | 302.6 (104.3) | 284.4 (90.1) | | 380.7 (127.3) | | 0.004* | |  |
| Blood creatinine AD (M6), µmol/L | 325.6 (132.8) | 311.6 (125.2) | | 385.6 (152.2) | | 0.031* | |  |
| Urea nitrogen BD (M3), mmol/L | 26.1 (6.5) | 25.9 (6.5) | | 27 (6.9) | | 0.644 | |  |
| Urea nitrogen BD (M6), mmol/L | 26.9 (7.2) | 26.9 (7.2) | | 26.5 (7.1) | | 0.841 | |  |
| Urea nitrogen AD (M3), mmol/L | 8.8 (3.1) | 8.2 (2.4) | | 11.4 (4.2) | | 0.006* | |  |
| Urea nitrogen AD (M6), mmol/L | 8.7 (3.1) | 8.4 (2.8) | | 10 (3.9) | | 0.165 | |  |
| Hemodialysis Equipment Treatment Parameters | | |  | |  | |  | |
| Treatment time (M3), hour | 3.9 (0.3) | 4 (0.3) | | 3.8 (0.3) | | 0.087 | |  |
| Treatment time (M6), hour | 3.9 (0.3) | 3.9 (0.3) | | 4 (0.3) | | 0.225 | |  |
| Blood flow (M3), mL/min | 142.8 (14.5) | 143.5 (15) | | 139.9 (11.9) | | 0.440 | |  |
| Blood flow (M6), mL/min | 143.4 (16.5) | 143.3 (16.2) | | 143.9 (18.4) | | 0.956 | |  |
| End conductivity (M3), mS/cm | 13.7 (0.2) | 13.7 (0.2) | | 13.8 (0.1) | | 0.192 | |  |
| End conductivity (M6), mS/cm | 13.8 (0.2) | 13.8 (0.2) | | 13.8 (0.2) | | 0.634 | |  |
| BC (M3), mS/cm | 30.9 (1) | 30.8 (0.8) | | 31.4 (1.6) | | 0.153 | |  |
| BC (M6), mS/cm | 31.1 (1.3) | 30.9 (0.7) | | 31.9 (2.4) | | 0.521 | |  |
| Dialysate flow rate (M3), mL/min | 486.5 (18.7) | 487 (20.3) | | 484.4 (9.1) | | 0.388 | |  |
| Dialysate flow rate (M6), mL/min | 486.5 (20.7) | 486.9 (21.4) | | 484.6 (18.1) | | 0.469 | |  |
| Dialysate temperature (M3), °C | 36.4 (0.2) | 36.4 (0.2) | | 36.3 (0.1) | | 0.143 | |  |
| Dialysate temperature (M6), °C | 36.4 (0.2) | 36.4 (0.1) | | 36.4 (0.2) | | 0.590 | |  |
| Reinfusion volume (M3), ml | 261.5 (66.5) | 268.7 (60.9) | | 230.7 (82.1) | | 0.061 | |  |
| Reinfusion volume (M6), ml | 271.3 (71.6) | 271.9 (75.8) | | 268.7 (51.6) | | 0.858 | |  |
| Dialyzer name (M3) |  |  | |  | | 0.787 | |  |
| Beltran lops12 | 41 (0.6) | 33 (0.6) | | 8 (0.6) | |  | |  |
| Beltran lops15 | 31 (0.4) | 25 (0.4) | | 6 (0.4) | |  | |  |
| Beltran HI-PS15 | 2 (0) | 2 (0) | | 0 (0) | |  | |  |
| Dialyzer name (M6) |  |  | |  | | 0.896 | |  |
| Beltran lops12 | 39 (0.5) | 32 (0.5) | | 7 (0.5) | |  | |  |
| Beltran lops15 | 33 (0.4) | 26 (0.4) | | 7 (0.5) | |  | |  |
| Beltran HI-PS15 | 1 (0) | 1 (0) | | 0 (0) | |  | |  |
| NIPRO SUREFLUX-130G | 1 (0) | 1 (0) | | 0 (0) | |  | |  |
| DCC (M3), mmol/L | 1.5 (0) | 1.5 (0) | | 1.5 (0) | | 0.923 | |  |
| DCC (M6), mmol/L | 1.5 (0) | 1.5 (0) | | 1.5 (0) | | 0.772 | |  |
| Ultrafiltration volume (M3), ml | 2056.9 (1017) | 1978.5 (1070.6) | | 2392.9 (675.3) | | 0.065 | |  |
| Ultrafiltration volume (M6), ml | 2130.4 (990.4) | 2006.3 (980.3) | | 2662.2 (878.4) | | 0.057 | |  |
| Venous pressure (M3), mm Hg | 101 (31.1) | 103.5 (31.7) | | 90.4 (26.6) | | 0.134 | |  |
| Venous pressure (M6), mm Hg | 107.7 (31.7) | 110.5 (31.1) | | 95.8 (32.8) | | 0.149 | |  |
| Arterial pressure (M3), mm Hg | -90.8 (48.7) | -89.6 (52.2) | | -95.9 (30.3) | | 0.619 | |  |
| Arterial pressure (M6), mm Hg | -106.8 (31.4) | -108.6 (31.5) | | -99.1 (31.4) | | 0.294 | |  |
| Actual MTP (M3), mm Hg | 113.6 (80.9) | 114.2 (88) | | 110.9 (40.6) | | 0.629 | |  |
| Actual MTP (M6), mm Hg | 123.4 (73.4) | 124.9 (79.8) | | 116.9 (35.7) | | 0.324 | |  |

Continuous variables are expressed as means (standard deviations). Categorical variables are expressed as counts (percentages). BD, before dialysis; AD, after dialysis; Total body water, based on Watson 1980 formula; BMI, body mass index; BSA, body surface area (Dubois formula); RRF, residual renal function; Kt/V, based on Daugirdas second generation logarithmically estimated single pool variable volume formula; CBP, Carbon dioxide binding power; SBP, systolic blood pressure; DBP, diastolic blood pressure; BC, bicarbonate conductivity; DCC, dialysate calcium concentration; MTP, maximum transmembrane pressure; M3, the third month after initiation of hemodialysis; M6, the six month after initiation of hemodialysis; Lower risk group, other reasons for hospitalization or non-hospitalization; Higher risk group, hospitalization for cardiovascular disease or all-cause death.

*P* indicates the significance level of the hypothesis test (Wilcoxon rank sum test for numeric variables and 𝜒2 test for categorical variables, two-tailed *p*-value < 0.05 was considered statistically significant).

**Table S3. Variance inflation factors for the exploratory saturated multivariable Cox model**

| **Predictor** | **Variance inflation factor** |
| --- | --- |
| Post dialysis weight (month 3) | 6.90 |
| Total body water (month 3) | 11.79 |
| Kt/V (month 3) | 2.35 |
| Kt/V (month 6) | 2.15 |
| Alkaline phosphatase (month 3) | 1.12 |
| Post dialysis creatinine (month 3) | 3.18 |
| Post dialysis urea nitrogen (month 3) | 3.79 |
| Bicarbonate conductivity (month 6) | 1.22 |
| Ultrafiltration volume (month 6) | 1.14 |

VIF values greater than 5 were observed for post dialysis weight and total body water at month 3, and several other dialysis adequacy and volume related parameters showed moderately elevated values, indicating substantial collinearity in the saturated model.

**Table S4. Exploratory saturated multivariable Cox model including all candidate predictors**

| **Predictor** | **Hazard ratio** | **95 percent confidence interval** | ***P* value** |
| --- | --- | --- | --- |
| Post dialysis weight (month 3) | 1.00 | 0.19-5.35 | 0.996 |
| Total body water (month 3) | 1.23 | 0.14-10.96 | 0.851 |
| Kt/V (month 3) | 0.85 | 0.32-2.26 | 0.743 |
| Kt/V (month 6) | 1.11 | 0.39-3.10 | 0.848 |
| Alkaline phosphatase (month 3) | 1.64 | 1.12-2.41 | 0.012 |
| Post dialysis creatinine (month 3) | 1.10 | 0.37-3.32 | 0.864 |
| Post dialysis urea nitrogen (month 3) | 1.72 | 0.55-5.44 | 0.354 |
| Bicarbonate conductivity (month 6) | 1.62 | 1.09-2.39 | 0.016 |
| Ultrafiltration volume (month 6) | 1.65 | 0.79-3.45 | 0.186 |

This saturated model includes all predictors that were statistically significant in univariable analyses, with additional adjustment for age and sex. Given the small number of events and the high degree of collinearity among dialysis adequacy and volume related parameters (Table S3), these estimates are presented only as exploratory and should be interpreted as hypothesis generating.
